# Effect of Toll‐like receptor 4 on depressive‐like behaviors induced by chronic social defeat stress

**DOI:** 10.1002/brb3.1525

**Published:** 2020-01-16

**Authors:** Ke Zhang, Wenjuan Lin, Juntao Zhang, Yawei Zhao, Xiaqing Wang, Mei Zhao

**Affiliations:** ^1^ CAS Key Laboratory of Mental Health Institute of Psychology Chinese Academy of Sciences Beijing China; ^2^ Department of Psychology University of Chinese Academy of Sciences Beijing China

**Keywords:** behavioral despair, chronic social defeat stress, depression, inflammation, TLR4

## Abstract

**Introduction:**

A growing body of evidence suggests that stress is an important factor in depression, and pro‐inflammatory cytokines contribute to the occurrence and development of depression in both animal models and human patients. Toll‐like receptor 4 (TLR4) has been shown to be a key innate immune pattern recognition receptor involved in the regulation of stress responses and inflammation. However, the exact effects of TLR4 on depressive‐like behaviors induced by chronic social defeat stress (CSDS) are not known.

**Methods:**

In this study, the effects of TLR4 on depressive‐like behaviors were investigated in an animal model of depression induced by CSDS. The depressive‐like behaviors were assessed by forced swimming test (FST), social interaction test (SIT), and light–dark box test (LDT). The protein expressions of TLR4 and tumor necrosis factor‐α (TNF‐α) in the hippocampus were measured using Western blotting.

**Results:**

We found that CSDS increased TLR4 protein levels in the hippocampus and induced behavioral despair in FST, social avoidance in SIT, and anxiety‐like behavior in LDT. Fluoxetine normalized the increased expression of TLR4 and reversed behavioral despair, social avoidance, as well as anxiety‐like behavior induced by CSDS. However, directly blocking TLR4, by using either TLR4 inhibitor TAK‐242 or knockout of TLR4, only inhibited behavioral despair, but not social avoidance or anxiety‐like behavior induced by CSDS.

**Conclusions:**

These results demonstrate a specific modulating role of TLR4 in behavioral despair induced by CSDS and suggest that TAK‐242 may be a beneficial treatment for patients with behavioral despair.

## Graphical abstract

These results demonstrate a specific modulating role of Toll‐like receptor 4 (TLR4) in behavioral despair induced by chronic social defeat stress.

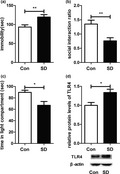

## 1 INTRODUCTION

Depression is a severe and chronic mental illness. It is characterized by sadness, anhedonia, low self‐esteem, fatigue, disturbed sleep or appetite, and poor concentration. Depression ranks within the top 4 “years lived with disability” and has one of the highest disease burdens (Vos et al., 2012). In recent years, the cytokine hypothesis has attracted much attention and many studies have shown that immune system activation plays an important role in the pathogenesis of depression (Raison, Capuron, & Miller, 2006). Patients with major depressive disorders exhibit increased circulating pro‐inflammatory cytokines, particularly tumor necrosis factor alpha (TNF‐α; Anisman & Hayley, 2012; Lichtblau, Schmidt, Schumann, Kirkby, & Himmerich, 2013). Additionally, a decrease in depressive symptoms is coupled with a normalization of pro‐inflammatory cytokines levels (Liu, Buisman‐Pijlman, & Hutchinson, 2014). Animal studies have also shown that pro‐inflammatory cytokines mediate depressive‐like behaviors induced by stress, and administration of cytokine‐inducer lipopolysaccharide (LPS) is sufficient to induce depressive‐like behaviors (Fleshner, Frank, & Maier, 2017; Tang, Lin, Pan, Guan, & Li, 2016).

The innate immune system senses pathogens through pattern recognition receptors (PRRs), and activation of PRRs induces downstream signaling pathways to mount appropriate immune responses (Liu & Ding, 2016). The PRRs that could recognize LPS is called Toll‐like receptor 4 (TLR4). TLR4, a kind of transmembrane protein, is a key innate immune pattern recognition receptor. TLR4 signaling is correlated with the modulation of the inflammatory mediator TNF‐α and increases the sensitivity of nociception (Chen et al., 2017). By recognizing exogenous pathogen‐associated molecular patterns (PAMPs) such as LPS, TLR4 triggers production of pro‐inflammatory cytokines, including TNF‐α, which are downstream signaling molecules of TLR4 (Liu et al., 2014). Inhibition of TLR4 or TLR4 deficiency blocked the elevation of cytokines in prefrontal cortex after stress (Gárate et al., 2013, 2014; Tramullas et al., 2014). In addition, TLR4‐knockout mice were resistant to learned helplessness induced by inescapable foot shock stress (Cheng et al., 2016). TLR4‐specific inhibitor reversed anhedonia induced by chronic unpredictable mild stress (Wang, Xu, Liu, Li, & Li, 2018). These studies imply potential roles of TLR4 in depression induced by stress. For better understanding the inflammatory processes associated with depression, systematic examination of the role of TLR4 in stress‐induced depression is needed.

Since the majority of stress stimuli in humans that lead to psychopathological changes are of social nature (Yan, Cao, Das, Zhu, & Gao, 2010), research on the consequences of social stress in experimental animal models is crucial. The chronic social defeat stress (CSDS) animal model is considered to be the most representative animal model for studying the consequences of social stress (Montagud‐Romero et al., 2018; Yanet al., 2010). In mice, CSDS induces depressive‐like behaviors and is becoming an increasingly popular model of depression (Hollis & Kabbaj, 2014). Although it has been demonstrated that only mice lacking TLR2 and TLR4 in combination (TLR2/4 double knockout) abolished CSDS‐induced social avoidance, neither TLR2 knockout nor TLR4 knockout alone could abolish CSDS‐induced social avoidance (Nie et al., 2018), the information about the effects of TLR4 on depressive‐like behaviors induced by social stress is limited. Thus, the purpose of the current study was to explore the role of TLR4 in depressive‐like behavior induced by CSDS. Fluoxetine was administrated to examine whether the efficacy of antidepressant drug was involved in the behavioral responses and the expression of TLR4 under CSDS condition. Then, both TLR4 inhibitor and TLR4 knockout mice were used to examine whether TLR4 had a direct effect on behavioral responses and the expression of TLR4 under CSDS condition. Since hippocampus is considered to be one of the most important areas involved in stress and depression, and our previous study has demonstrated that TNF‐α is a common risk factor in depressive disorders induced by both stress and inflammation (Guan, Lin, & Tang, 2015), expression of hippocampal TLR4 as well as TNF‐α was examined in the current study.

## 2 MATERIALS AND METHODS

### 2.1 Animals

Wild‐type male C57BL/6 Mice were purchased from Beijing Huafukang Bioscience Co. LTD. TLR4‐knockout (KO) mice (B6.B10ScN‐Tlr4^lps‐del^/JthJ) were generously provided by Prof. Xiaodong Shi (Institute of Biophysics of the Chinese Academy of Sciences). Wild‐type (WT) C57BL/6 Mice and TLR4‐KO mice were housed in groups of four per cage in stainless steel cages, in a temperature‐ and humidity‐controlled room (22 ± 1°C; 40%–60% humidity) with a 12:12 dark/light cycle (lights on at 07:00 a.m.; off at 7:00 p.m.). Mice were given free access to food and water throughout the experiment. All mice were 9–11 weeks old at the start of all the experiments. The experimental procedures were approved by the Institutional Review Board of the Institute of Psychology, Chinese Academy of Sciences, and were consistent with the National Institutes of Health Guide for the Care and Use of Laboratory Animals.

### 2.2 Drug administration

TAK‐242 (Cat#HY‐11109), a specific inhibitor of TLR4 (Takashima et al., 2009), was obtained from MedChemExpress. TAK‐242 was dissolved in DMSO and then diluted in sterile water. The final concentration of DMSO was 5%. Fluoxetine (Cat#HY‐B0102A) was obtained from MedChemExpress. Fluoxetine was dissolved in 0.9% sterile saline.

### 2.3 Experimental procedures

#### 2.3.1 Experiment 1: The effects of CSDS on depressive‐like behaviors and the protein expression of TLR4 in the hippocampus

Experiment 1 examined whether TLR4 was involved in the occurrence of depressive‐like behaviors induced by CSDS. WT mice were randomly assigned to the control (Con) group and the stress (SD) group. For SD group, a C57 intruder mouse was subjected to physical and sensory contact with a novel CD‐1 aggressor mouse each day for a total of 10 days. Control mice were housed in equivalent cages but with animals of the same strain without direct interaction. Twenty‐four hours after CSDS, C57 mice were examined with behavioral tests. Seventy‐two hours after CSDS, mice were decapitated and the hippocampus was isolated for Western blot analysis.

#### 2.3.2 Experiment 2: The effects of fluoxetine on CSDS‐induced depressive‐like behaviors and the altered expression of TLR4 as well as TNF‐α in the hippocampus

Experiment 2 examined whether TLR4 was involved in antidepressive effects of fluoxetine. WT mice were randomly assigned to the control/saline group (Con/sal), stress/saline group (SD/sal), control/fluoxetine group (Con/FLX), and stress/fluoxetine group (SD/FLX). Fluoxetine (20 mg kg^−1^ day^−1^) or saline was administrated (i.p.) prior to and concurrent with CSDS for 21 days (Han, Lee, & Leem, 2015; Lehmann, Geddes, Lee, & Herkenham, 2013; Talbot et al., 2016). Twenty‐four hours after CSDS, C57 mice were examined with behavioral tests. Seventy‐two hours after CSDS, mice were decapitated and the hippocampus was isolated for Western blot analysis.

#### 2.3.3 Experiment 3: The effects of TLR4 inhibitor (TAK‐242) on CSDS‐induced depressive‐like behaviors and the altered expression of TLR4 as well as TNF‐α in the hippocampus

Experiment 3 examined whether TLR4 had a direct effect on CSDS‐induced depressive‐like behaviors through TLR4 inhibitor TAK‐242. WT mice were randomly assigned to 4 groups: control/vehicle group (Con/veh), stress/vehicle group (SD/veh), control/TAK‐242 group (Con/TAK), and stress/TAK‐242 group (SD/TAK). Twenty‐four hours after CSDS, C57 mice were examined with behavioral tests. TAK‐242 (10 mg/kg body weight) or saline was injected intraperitoneally once 1 hr before behavioral test (Tramullas et al., 2014). For another four separated groups of mice, 72 hr after CSDS, C57 mice were decapitated without behavioral testes. TAK‐242 was injected intraperitoneally (10 mg/kg body weight) 1 hr before decapitation. Hippocampus was isolated for Western blot analysis.

#### 2.3.4 Experiment 4: The effects of knockout of TLR4 on CSDS‐induced depressive‐like behaviors and the altered expression of TNF‐α in the hippocampus

Experiment 4, by using TLR4‐KO mice, further examined TLR4's direct effect on CSDS‐induced depressive‐like behaviors. WT mice were randomly assigned to two groups: WT/control group (WT/Con) and the WT/stress group (WT/SD). The TLR4‐KO mice were randomly assigned to two groups: knockout/control group (KO/Con) and knockout/stress group (KO/SD). Twenty‐four hours after CSDS, C57 mice were examined with behavioral tests. For another four separated groups of mice (WT/Con, WT/SD, KO/Con, and KO/SD), 72 hr after CSDS, mice were decapitated without behavioral testes, and the hippocampus was isolated for Western blot analysis.

### 2.4 Chronic social defeat stress

Chronic social defeat stress was induced using a procedure similar to one reported previously (Li et al., 2018). For stress group, a C57BL/6 (C57) intruder mouse was subjected to a novel CD‐1 aggressor mouse for 5–10 min each day for a total of 10 days. Every day after physical contact, the intruder C57 and CD‐1 mice were separated into the opposite side of the social defeat cage, which was divided by a plastic wall containing holes in the middle to allow for sensory contact during the subsequent 24 hr. Control mice were housed in equivalent cages but with animals of the same strain without direct interaction. Following the final sensory contact, C57 mice were singly housed for 24 hr and then examined with behavioral tests.

### 2.5 Behavioral tests

#### 2.5.1 Social interaction test

The social interaction test (SIT) was performed to assess social avoidance of the mice (Tramullas et al., 2014), which is a core symptom of depressive‐like behaviors. As described previously (Li et al., 2018), an experimental or control C57 mouse was placed in an open field (42 × 42 cm) with a small empty cage at one end. Their movements were then automatically monitored and recorded by the Video Detecting System (Anilab) for 2.5 min in the absence of the novel CD‐1 mouse. Then, the CD‐1 mouse was introduced into the cage and the procedure was repeated. The social interaction ratio was calculated as the time spent in an interaction zone near the novel CD‐1 mouse divided by the time spent in the zone near the empty cage.

#### 2.5.2 Light–dark box test

The light–dark box test (LDT) was performed to assess the anxiety‐like status of the mice (Bourin & Hascoët, 2003), which is depressive‐like behaviors' concomitant symptom. A plexiglass box was divided into two compartments. The bright chamber made of transparent plastic was connected to the dark compartment which was black and opaque. Each animal was allowed to explore the box freely for 5 min. The time spent in the light area was recorded.

#### 2.5.3 Forced swimming test

The forced swimming test (FST) was used to assess behavioral despair, a core symptom of depressive‐like behaviors (Porsolt, Le Pichon, & Jalfre, 1977). Mice were placed into a glass cylinder filled with water for 6 min. The mice were judged immobile when they ceased struggling and remained floating motionless in the water (without any vertical or horizontal movements), performing only the movements necessary to keep their heads above the water level. An increased duration of immobility in this test is used conventionally as an index of behavioral despair.

### 2.6 Western blotting

Mice were decapitated 24 hr after the behavioral tests, and brains were rapidly removed. According to the mouse brain map (Paxinos & Franklin, 2004), the hippocampus was dissected on ice and placed into liquid nitrogen to be frozen and then stored at −80°C until use. Proteins from hippocampus were dissolved in RIPA lysis buffer (CW Biotech), and their concentration was measured using BCA Protein Assay Kit (CW Biotech). Total proteins were separated by sodium dodecyl sulfate–polyacrylamide gel electrophoresis (SDS‐PAGE) in reducing conditions using 12% or 8% acrylamide gels and analyzed by immunoblotting using anti‐TLR4 (1:1,000; ab13867; Abcam), anti‐TNF‐α (1:1,000; ab66579; Abcam), and anti‐β‐actin (1:10,000, #3700; CST) as primary antibodies. Band intensity was semi‐quantitatively analyzed using the Quantity One software (Bio‐Rad), and the expression level of TLR4 and TNF‐α was normalized to that of β‐actin.

### 2.7 Statistical analysis

All data were expressed as the mean ± *SEM*. Statistical analyses were performed with the SPSS software. Unpaired Student's *t* test was used in analysis of results of experiment 1. Two‐way analysis of variance (ANOVA) followed by a Bonferroni post hoc test was used in analysis of results of experiment 2, experiment 3, and experiment 4. Statistical significance was set at *p* < .05.

## 3 RESULTS

### 3.1 CSDS induced depressive‐like behaviors and increased TLR4 expression in hippocampus

**Figure 1:**
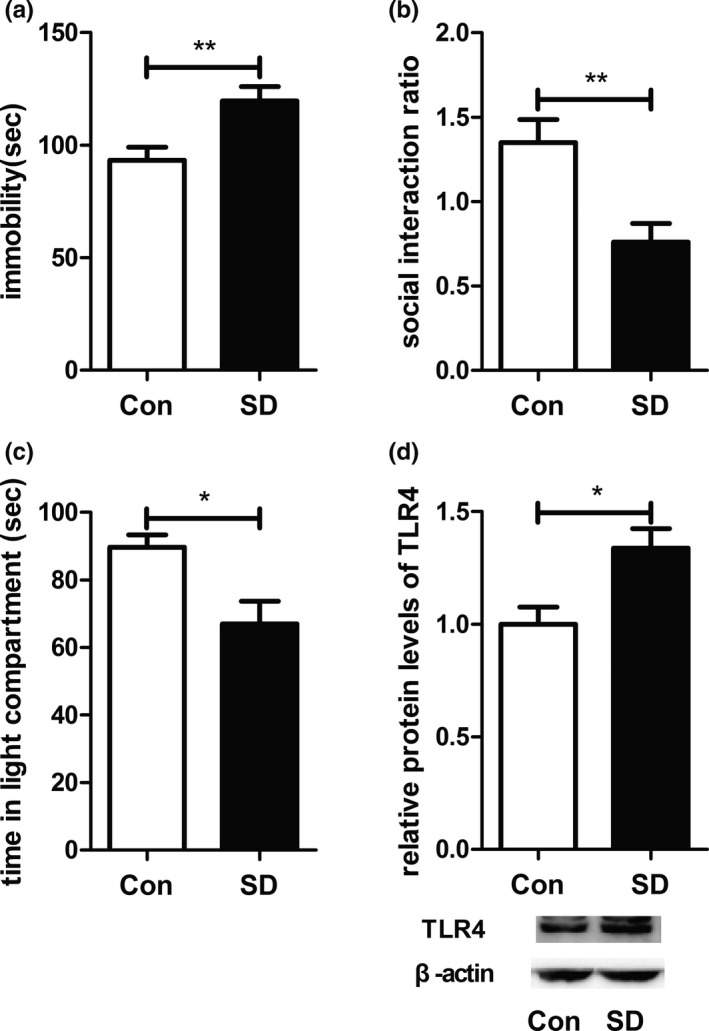
Effects of chronic social defeat stress on depressive‐like behaviors and protein expression of TLR4 in hippocampus. (a) Immobility duration in the forced swimming test. (b) Social interaction ratio in social interaction test. (c) Time in the light area in light–dark box test. (d) Protein levels of TLR4 in hippocampus from Con and SD groups. Con group (*n* = 8), SD group (*n* = 8). *p < .05; **p < .01. Con, control; SD, stress; TLR4, Toll‐like receptor 4

After 10 days' CSDS, FST, SIT, and LDT were performed to assess the depressive status of the mice. Compared to the control group, the stress group exhibited a longer immobility duration in the FST (*t*_14_ = 3.061, *p* < .01; Figure 1a), showed significant decrease in the social interaction ratio during the SIT (*t*_14_ = 3.346, *p* < .01; Figure 1b), and spent less time in the light area of LDT (*t*_14_ = 2.971, *p* < .05; Figure 1c). Concurrently, expression of the hippocampal TLR4 proteins increased significantly in the stress group (*t*_14_ = 2.962, *p* < .05; Figure 1d). The results suggested that TLR4 was involved in the occurrence of depressive‐like behaviors induced by CSDS.

### 3.2 Fluoxetine reversed CSDS‐induced depressive‐like behaviors and the increased expression of TLR4 as well as TNF‐α in the hippocampus

To study whether TLR4 was also involved in antidepressive effects of fluoxetine, fluoxetine was administrated before and during CSDS, and then, behavioral responses and expression of hippocampal TLR4 as well as TNF‐α were examined.

**Figure 2:**
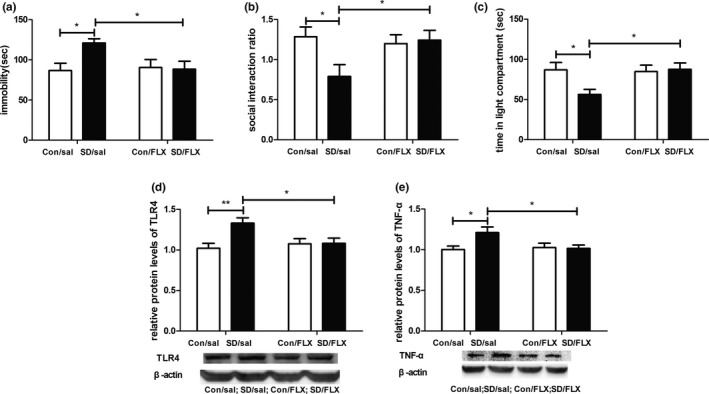
Effects of fluoxetine on chronic social defeat stress‐induced depressive‐like behaviors and increased protein expression of TLR4 as well as TNF‐α in hippocampus. (a) Immobility duration in the forced swimming test. (b) Social interaction ratio in social interaction test. (c) Time in the light area in light–dark box test. (d) Protein levels of TLR4 in hippocampus from four groups. (e) Protein levels of TNF‐α in hippocampus from four groups. *n* = 7 per group. *p < .05, **p < .01. Con, control; FLX, fluoxetine; sal, saline; SD, stress; TLR4, Toll‐like receptor 4; TNF‐α, tumor necrosis factor‐α

For FST, a two‐way ANOVA revealed a significant effect of stress × fluoxetine interaction (*F*_1,24_ = 4.429, *p* < .05) on immobility duration. Post hoc analyses revealed that immobility duration of mice in the SD/sal group was longer than that of mice in the Con/sal group (*p* < .05). The immobility duration of mice in the SD/FLX group was significantly decreased compared to that of mice in the SD/sal group (*p* < .05; Figure 2a). For open‐field test, a two‐way ANOVA did not reveal a significant effect of stress × fluoxetine interaction (*F*_1,24_ = 1.91, *p* > .05) on total distance traveled. Unpaired Student's *t* test showed that the total distance traveled of mice in the SD/sal group was not smaller than that of mice in the Con/sal group (*t*_12_ = 1.82, *p* > .05), and the total distance traveled of mice in the SD/sal group was not smaller than that of mice in the SD/FLX group (*t*_12_ = 1.90, *p* > .05).

For SIT, a two‐way ANOVA revealed a significant effect of stress × fluoxetine interaction (*F*_1,24_ = 4.511, *p* < .05) on social interaction ratio. Post hoc analyses revealed that the social interaction ratio of mice in the SD/sal group was lower than that of mice in the Con/sal group (*p* < .05). The social interaction ratio of mice in the SD/FLX group was significantly increased compared to that of mice in the SD/sal group (*p* < .05; Figure 2b).

For LDT, a two‐way ANOVA revealed a significant effect of stress × fluoxetine interaction (*F*_1,24_ = 4.35, *p* < .05) on time spent in the light area. Post hoc analyses revealed that time spent in the light area of mice in the SD/sal group was significantly decreased compared to that of mice in the Con/sal group (*p* < .05). The time spent in the light area of mice in the SD/FLX group was significantly increased compared to that of mice in the SD/sal group (*p* < .05; Figure 2c).

For TLR4 protein level, a two‐way ANOVA revealed a significant effect of stress (*F*_1,24_ = 5.974, *p* < .05) and stress × fluoxetine interaction (*F*_1,24_ = 5.545, *p* < .05). Post hoc analyses revealed that hippocampal TLR4 protein levels of mice in the SD/sal group were higher than that of mice in the Con/sal group (*p* < .01). The hippocampal TLR4 protein levels of mice in the SD/FLX group were significantly decreased compared to that of mice in the SD/sal group (*p* < .05; Figure 2d).

For TNF‐α protein level, a two‐way ANOVA revealed a significant effect of stress × fluoxetine interaction (*F*_1,24_ = 4.388, *p* < .05). Post hoc analyses revealed that hippocampal TNF‐α protein levels of mice in the SD/sal group were higher than that of mice in the Con/sal group (*p* < .05). The hippocampal TNF‐α protein levels of mice in the SD/FLX group were significantly decreased compared to that of mice in the SD/sal group (*p* < .05; Figure 2e).

Taken together, these findings showed that fluoxetine treatment significantly reversed CSDS‐induced depressive‐like behaviors and the increased expression of TLR4 as well as TNF‐α in the hippocampus. These findings suggested that TLR4 was also involved in antidepressive effects of fluoxetine.

### 3.3 TLR4 inhibition reversed CSDS‐induced behavioral despair and the increased expression of TLR4 as well as TNF‐α in the hippocampus

To examine TLR4's direct effect on depressive‐like behaviors induced by CSDS, the effect of TAK‐242, a TLR4 inhibitor, on behavioral responses and the expression of TLR4 as well as TNF‐α induced by CSDS were examined.

**Figure 3:**
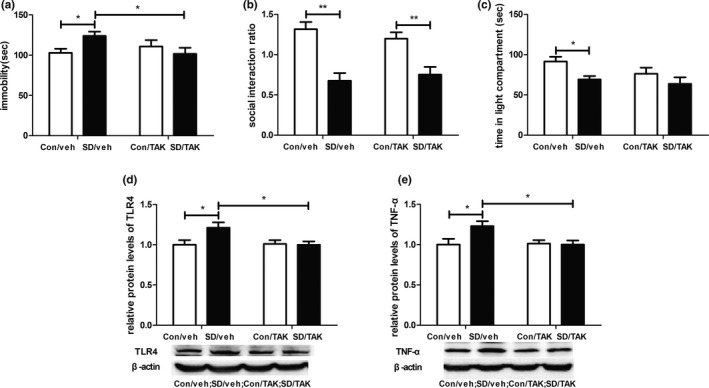
Effects of TLR4 inhibitor (TAK‐242) on chronic social defeat stress‐induced depressive‐like behaviors and increased expression of TLR4 as well as TNF‐α in hippocampus. (a) Immobility duration in the forced swimming test. (b) Social interaction ratio in social interaction test. (c) Time in the light area in light–dark box test. (d) Protein levels of TLR4 in hippocampus from four groups. (e) Protein levels of TNF‐α in hippocampus from four groups. *n* = 7–10 per group. *p < .05, *p* < .01. Con, control; SD, stress; TAK, TAK‐242; TLR4, Toll‐like receptor 4; TNF‐α, tumor necrosis factor‐α; veh, vehicle

For FST, a two‐way ANOVA revealed a significant effect of stress × TAK‐242 interaction (*F*_1,33_ = 5.409, *p* < .05) on immobility duration. Post hoc analyses revealed that immobility duration of mice in the SD/veh group was longer than that of mice in the Con/veh group (*p* < .05). The immobility duration of mice in the SD/TAK group was significantly decreased compared to that of mice in the SD/veh group (*p* < .05; Figure 3a).

For SIT, a two‐way ANOVA revealed a significant effect of stress (*F*_1,33_ = 35.5, *p* < .001) on social interaction ratio. Post hoc analyses revealed that the social interaction ratio of mice in the SD/veh group was lower than that of mice in the Con/veh group (*p* < .01), and the social interaction ratio of mice in the SD/TAK group was lower than that of mice in the Con/TAK group (*p* < .01; Figure 3b).

For LDT, a two‐way ANOVA revealed a significant effect of stress (*F*_1,33_ = 7.114, *p* < .05) on time spent in the light area. Post hoc analyses revealed that time spent in the light area of mice in the SD/veh group was significantly decreased compared to that of mice in the Con/veh group (*p* < .05). The difference between time spent in the light area of mice in the SD/veh group and that of mice in the SD/TAK group was not statistically significant (Figure 3c).

For TLR4 expression, a two‐way ANOVA revealed a significant effect of stress × TAK‐242 interaction (*F*_1,24_ = 4.323, *p* < .05). Post hoc analyses revealed that hippocampal TLR4 protein levels of mice in the SD/veh group were higher than that of mice in the Con/veh group (*p* < .05). The hippocampal TLR4 protein levels of mice in the SD/TAK group were significantly decreased compared to that of mice in the SD/veh group (*p* < .05; Figure 3d).

A two‐way ANOVA also revealed a significant effect of stress × TAK‐242 interaction (*F*_1,24_ = 4.442, *p* < .05) on TNF‐α protein level. Post hoc analyses revealed that hippocampal TNF‐α protein level of mice in the SD/veh group was higher than that of mice in the Con/veh group (*p* < .05). The hippocampal TNF‐α protein levels of mice in the SD/TAK group were significantly decreased compared to that of mice in the SD/veh group (*p* < .05; Figure 3e).

Taken together, these data indicated that acute injection of TLR4 inhibitor just prior to behavioral test could block behavioral despair, but not social avoidance and anxiety‐like behavior induced by CSDS. And TLR4 inhibitor reversed CSDS‐induced increased expression of TLR4 and TNF‐α in the hippocampus.

### 3.4 Knockout of TLR4 blocked CSDS‐induced behavioral despair and the increased expression of TNF‐α in the hippocampus

The effect of knockout of TLR4 on behavioral responses and expression of TNF‐α induced by CSDS were further examined to verify the roles of TLR4 in depressive‐like behavior induced by CSDS.

**Figure 4:**
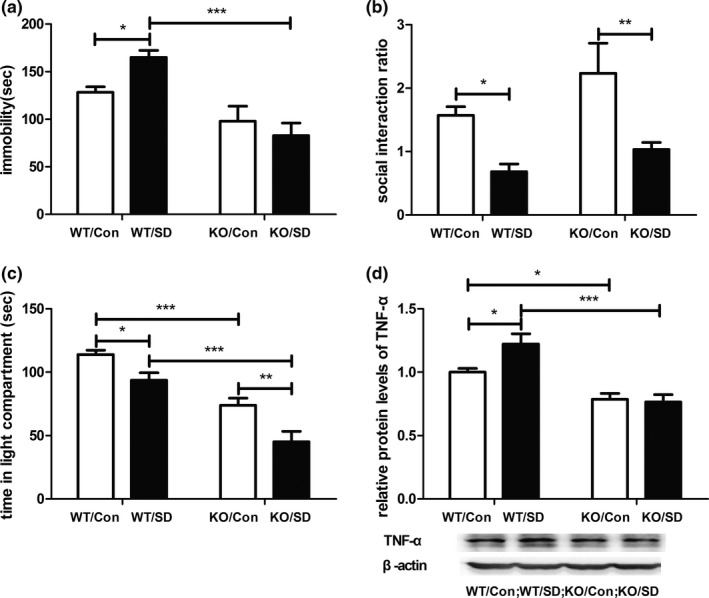
Effects of knockout of TLR4 on chronic social defeat stress‐induced depressive‐like behaviors and increased protein expression of TNF‐α in hippocampus. (a) Immobility duration in the forced swimming test. (b) Social interaction ratio in social interaction test. (c) Time in the light area in light–dark box test. (d) Protein levels of TNF‐α in hippocampus from four groups. *n* = 6–10 per group. *p < .05; **p < .01; ***p < .001. Con, control; KO, knockout; SD, stress; TNF‐α, tumor necrosis factor‐α; TLR4, Toll‐like receptor 4; WT, wild‐type

For FST, a two‐way ANOVA revealed a significant effect of genotype (*F*_1,28_ = 25.813, *p* < .001) and a significant effect of stress × genotype interaction (*F*_1,28_ = 5.493, *p* < .05) on immobility duration. Post hoc analyses revealed that immobility duration of mice in the WT/SD was longer than that of mice in the WT/Con (*p* < .05). The difference between the immobility duration of mice in the KO/Con and that of mice in the KO/SD was not statistically significant. The immobility duration of mice in the WT/SD group was longer than that of mice in the KO/SD group (*p* < .001; Figure 4a).

For SIT, a two‐way ANOVA revealed a significant effect of stress (*F*_1,31_ = 15.287, *p* < .001) on social interaction ratio. Post hoc analyses revealed that the social interaction ratio of mice in the WT/SD group was lower than that of mice in the WT/Con group (*p* < .05), and the social interaction ratio of mice in the KO/SD group was lower than that of mice in the KO/Con group (*p* < .01; Figure 4b).

For LDT, a two‐way ANOVA revealed significant effect of stress (*F*_1,30_ = 17.801, *p* < .001) and significant effect of genotype (*F*_1,30_ = 58.351, *p* < .001) on time spent in the light area. Post hoc analyses revealed that time spent in the light area of mice in the WT/SD group was significantly decreased compared to that of mice in the WT/Con group (*p* < .05). Time spent in the light area of mice in the KO/SD group was significantly decreased compared to that of mice in the KO/Con group (*p* < .01). Time spent in the light area of mice in the KO/Con group was significantly decreased compared to that of mice in the WT/Con group (*p* < .001). Time spent in the light area of mice in the KO/SD group was significantly decreased compared to that of mice in the WT/SD group (*p* < .001; Figure 4c).

A two‐way ANOVA revealed a significant effect of genotype (*F*_1,22_ = 33.714, *p* < .001) and stress × genotype interaction (*F*_1,22_ = 4.45, *p* < .05) on TNF‐α protein level. Post hoc analyses revealed that hippocampal TNF‐α protein levels of mice in the WT/SD group were higher than that of mice in the WT/Con group (*p* < .05). The difference between the hippocampal TNF‐α protein levels of mice in the KO/Con group and that of mice in the KO/SD group was not statistically significant. The hippocampal TNF‐α protein levels of mice in the WT/Con group were higher than that of mice in the KO/Con group (*p* < .05). The hippocampal TNF‐α protein levels of mice in the WT/SD group were higher than that of mice in the KO/SD group (*p* < .001; Figure 4d).

Taken together, these data indicate that knockout of TLR4 could block behavioral despair, but not social avoidance and anxiety‐like behavior induced by CSDS. And knockout of TLR4 could prevent CSDS‐induced increased expression of TNF‐α in the hippocampus.

## 4 DISCUSSION

In the present study, we found that CSDS resulted in depressive‐like behaviors including increased immobility duration in FST, decreased social interaction ratio in SIT, and decreased time spent in the light area in LDT, accompanied by increased TLR4 protein level in hippocampus. Hippocampus is considered to be one of the most important areas involved in stress and depression. For example, neuroinflammation, apoptosis, reduced neurogenesis, and dysfunction of microglia in hippocampus are associated with depression (Kubera, Obuchowicz, Goehler, Brzeszcz, & Maes, 2011; Tang et al., 2016; Zhang, Zhang, & You, 2018; Zhao et al., 2019). In this study, by using the antidepressant fluoxetine, TLR4 inhibitor, and TLR4 knockout mice, our results suggest that hippocampal TLR4 is involved in behavioral despair induced by CSDS.

Fluoxetine is a widely used antidepressant which could reverse depressive‐like and anxiety‐like behaviors (Habib, Shaker, El‐Gayar, & Aboul‐Fotouh, 2015; Lehmann et al., 2013; Wang et al., 2014). But there is limited evidence about whether TLR4 is involved in the antidepressant effect of fluoxetine. Our findings showed that fluoxetine not only reversed behavioral despair, social avoidance, and anxiety‐like behavior, but also normalized increased expression of hippocampal TLR4 induced by CSDS. Our finding suggests that hippocampal TLR4 was involved in pharmacological mechanism of the antidepressant action of fluoxetine in depressive‐like behaviors induced by CSDS.

Because fluoxetine has been commonly thought to act via inhibiting 5‐hydroxytraptamine reuptake in the central nervous system (Cipriani et al., 2005), the possibility that TLR4 contributes to the modulation of the depressive‐like behaviors induced by CSDS needs further examination. To clarify direct roles of TLR4 on behavior and molecular changes induced by CSDS, TLR4 inhibitor (TAK‐242) was used. Our results showed that one injection of TAK‐242 prior to behavioral test could block behavioral despair but not social avoidance and anxiety‐like behavior, suggesting that TLR4 is effective to modulate despair phenotype induced by CSDS.

The finding that TLR4 was effective to modulate despair phenotype induced by CSDS was further demonstrated by TLR4 knockout mice. Consistent with the TLR4 inhibitor's effect on behavioral despair, we found that knockout of TLR4 could reverse the behavioral despair indexed by the immobility duration but not social avoidance and anxiety‐like behavior induced by CSDS. Our results about the effect of knockout of TLR4 on social interaction ratio in SIT were in agreement with the previous study (Tramullas et al., 2014). It has been reported that only mice lacking TLR2 and TLR4 in combination (TLR2/4 double knockout) abolished CSDS‐induced social avoidance. Neither TLR2 knockout nor TLR4 knockout alone could abolish CSDS‐induced social avoidance (Nie et al., 2018). Combined with our results, TLR4, in combination with TLR2, may participate in modulation of social avoidance, while TLR4 alone only modulates behavioral despair.

As to the anxiety‐like behavior, we found that unstressed TLR4‐KO mice spent less time in light area in the LDT compared to unstressed WT mice, which was supported by a recent study in which mice lacking TLR4 had a robust anxiety‐like phenotype (Femenia, Qian, Arentsen, Forssberg, & Heijtz, 2018).

It is well known that TLR4 triggers production of pro‐inflammatory cytokines (Liu et al., 2014). Since our previous study has demonstrated that TNF‐α is a common risk factor in depressive disorders induced by both stress and inflammation (Guan et al., 2015), we further investigated whether antidepressant effect of TLR4 is via inhibition of TNF‐α production. We found that injection of TAK‐242 could prevent CSDS‐induced increased expression of both TLR4 and TNF‐α in hippocampus. This result is supported by previous reports in which TAK‐242 could prevent the elevated TLR4 in frontal cortex induced by restraint stress and the increased TNF‐α and IL‐6 in serum induced by LPS (Gárate et al., 2014; Sha et al., 2007; Wang et al., 2016; Yu, Cheng, Du, Huang, & Dong, 2013). Our result showed that knockout of TLR4 caused lower basal TNF‐α expression, and the result that knockout of TLR4 prevented CSDS‐induced increased expression of TNF‐α in hippocampus was also consistent with the findings that knockout of TLR4 could prevent increased TNF‐α in hippocampus induced by inescapable foot shocks (Cheng et al., 2016), and deficiency of *Tlr4* gene could prevent the upregulation of pro‐inflammatory cytokines in prefrontal cortex induced by restraint stress (Gárate et al., 2013). Recently, studies indicate that after exposure to stress, microglial cells were induced into the M1 phenotype, which was associated with upregulating pro‐inflammatory cytokines and the onset of psychiatric disorders (Zhang et al., 2017). TLR4 deficiency could induce microglial polarization toward the M2 phenotype and downregulating pro‐inflammatory cytokines, which may contribute to the prevention of psychiatric disorders (Yao et al., 2017; Zhang et al., 2018; Zhao et al., 2019).

In conclusion, the results showed that CSDS increased the TLR4 protein level in hippocampus and induced behavioral despair, social avoidance, and anxiety‐like behavior. Fluoxetine blocked the increased expression of TLR4 and reversed behavioral despair, social avoidance, and anxiety‐like behavior induced by CSDS. Either acute injection of TLR4 inhibitor or knockout of TLR4 prevented the CSDS‐induced behavioral despair, but not social avoidance and anxiety‐like behavior. TLR4 inhibitor normalized the increased expression of TLR4 as well as TNF‐α induced by CSDS, and knockout of TLR4 also prevented upregulation of TNF‐α protein induced by CSDS. Collectively, our results demonstrated a specific role of TLR4 in behavioral despair induced by CSDS and suggested that TAK‐242, a small molecule that selectively inhibits TLR4‐mediated signaling, may be a beneficial treatment for patients with behavioral despair.

## CONFLICT OF INTEREST

All authors have no conflicts of interest to declare.

## Data Availability

The data that support the findings of this study are available from the corresponding author upon reasonable request.
